# Notes and News

**Published:** 1894-01-27

**Authors:** 


### NOTES AND NEWS.

COLLECTED AND COMMUNICATED.

THE law relating to the notification of infectious diseases in France, which came into force at the end of the year, includes typhoid and typhus fevers, small-pox, scarlet fever, diphtheria, cholera, dysentery, puerperal fever, and ophthalmia in the newly-born. The system provides medical men with post-cards, with particulars to be filled in. By a duplicate system numbers are used specifying some particulars the medical man desires to convey, and his identification is also provided for by numbering the cards, without necessitating his signature.

IT is becoming generally recognised by the public and impressed by the medical faculty that much contagion could be prevented by the habitual boiling of all milk before use. It seems, however, that, as in the case of the infection of phthisis, the example official initiative is set by the newer continents. At a recent meeting of the Victorian Branch of the British Medical Association a resolution was passed recommending householders to boil their milk, and suggesting that all purveyors of milk should be compelled by law to produce on demand by customers a certificate from the local officer of health that their business arrangements are sanitary.

THE want of provision for scarlet fever cases at Bradford has fallen very heavily on the Bradford Infirmary, and has proved a great danger to the town. For some time cases of fever have appeared at the hospital, and to prevent the spread of the disease amongst the other patients the sufferers were sent to their own homes. Nevertheless the infection was not stamped out, and eventually spread to the nurses. Arrangements were then made to turn the nurses' home into an isolation hospital for nurses and patients, and thus check a further outbreak. The petition that has been made for proper provision for scarlet fever at Bradford has been met so far by a reply from the sanitary authorities that they are using every endeavour with a view to meeting the difficulty.

IN America much attention is drawn in the Press to the number of accidents resulting from football. Statistics are systematically collected, and the number of casualties quoted. In November six fatalities are recorded, besides a number of accidents of a serious nature.

IT is very encouraging to observe that amongst the medical charities a spirit of emulation and aspiration towards efficiency is visibly growing. In "The Uniform System of Accounts," published by the Scientific Press, we were able to comment in favourable terms on the excellent system of returns which prevailed at the Royal Edinburgh Infirmary. The Chairman of that institution now calls our attention to the fact that our criticism has proved most encouraging, and has been embodied in the report of the infirmary for the present year.

ATTENTION at present is being directed to the expenditure on surgical instruments at the Addenbrooke's Hospital, Cambridge. The expenses under this heading in 1887 were compared to those of 1893, and found to be so widely different as to provoke comment. The explanation now given by Dr. Humphrey shows that whilst the sum of £66 expended in 1887 was an unusual and minimum one, that of £160 in 1893 was not by any means unprecedented, or, indeed, above the average. It appearing that so many items being included under the heading of surgical instruments is misleading, steps will be taken to redistribute the expenditure, and nurses and surgeons will be especially requested to exercise economy in the use of dressings.

AN extraordinary notion of hospital etiquette was recently revealed in Dublin. The death of a woman who had been refused admission to several hospitals in the city caused an inquiry to be raised. In the course of this inquiry it was stated that the house surgeon of the second hospital at which admission was desired refused it on the ground that etiquette did not allow one hospital to admit patients refused by another. It is satisfactory that the authorities of the hospital in question strongly condemned the action of their house surgeon, and provided that such procedure should be prevented in the future. Frequent mistakes as to the rejection of cases which occur at hospitals would be avoided were the junior staff more carefully instructed in this matter, and accommodation for casualty patients always provided.

AN instance of heroic devotion to the work he had undertaken has been recently recorded of the vicar of a crowded parish. Finding that without the assistance of an additional curate he could not discharge his duties as he desired to his parishioners, the Rev. J. B. Mylius, the vicar of All Saints, Hatcham Park, undertook the duties of chaplain to the South-Eastern Hospital that he might procure the necessary funds for further help. For nearly three years he performed his duties to the parish and hospital with devotion. A short time ago he fell a victim to the fever, and passed away at the early age of thirty-two. Such lives are not lost, though their span is short. They strengthen and encourage the weak-hearted, and their example is doubly impressive where regret remains for the briefness of their sojourn amongst us.

